# Chrono-Immune Neuropathy During PD-1 Blockade: A Report of a Fatal Case and Hypothesis on Circadian Modulation of Neurotoxicity

**DOI:** 10.7759/cureus.99703

**Published:** 2025-12-20

**Authors:** Ivan Bivolarski

**Affiliations:** 1 Medical Oncology, Integrated Oncology Centre Burgas, Burgas, BGR

**Keywords:** chronotherapy, circadian rhythm, immune checkpoint inhibitor, melanoma, neuropathic pain treatment, neurotoxicity

## Abstract

Immune checkpoint inhibitors can cause a wide spectrum of immune-related adverse events, including rare but severe neurological complications. This report describes a fatal case of progressive immune-mediated neuropathy in a melanoma patient treated with PD-1 blockade, in whom worsening neuropathic pain and functional decline coincided with irregular, non-aligned timing of immunotherapy infusions. PET-CT imaging, MRI, and laboratory testing excluded metastatic, infectious, metabolic, and paraneoplastic causes. Longitudinal evaluation revealed fluctuations in inflammatory indices, including neutrophil-to-lymphocyte ratio (NLR), platelet-to-lymphocyte ratio (PLR), and systemic immune-inflammation index (SII), with peaks occurring shortly after late-afternoon infusions. Earlier infusions were associated with fewer symptoms. Although causality cannot be established from a single case, this temporal association raises the possibility that circadian desynchronization may amplify immune activation and contribute to neurotoxicity. Based on these observations, the concept of “Chrono-Immune Neuropathy” is proposed, suggesting that disrupted temporal regulation of treatment may influence the severity of neurologic immune-related adverse events. This case highlights the potential importance of consistent, circadian-aware scheduling in immunotherapy and supports further investigation into time-of-day-sensitive treatment strategies aimed at reducing toxicity and improving patient outcomes.

## Introduction

Immune checkpoint inhibitors have transformed the management of advanced melanoma and many other solid tumors, but they can also produce a wide range of immune-related adverse events, including rare yet potentially devastating neurological complications [1,2]. Immune-mediated neuropathies remain poorly understood because their symptoms are nonspecific, biomarker correlates are unreliable, and radiological findings are frequently absent. Proposed mechanisms include autoreactive T-cell activation, cytokine-driven neuroinflammation, and disruption of the blood-brain barrier, yet predictive factors remain elusive [3]. In clinical practice, these toxicities often emerge unpredictably and may progress rapidly despite prompt discontinuation of therapy. Early recognition, therefore, depends on careful integration of clinical, laboratory, and temporal patterns rather than on any single diagnostic marker.

Circadian biology has emerged as a major regulator of immune function, influencing lymphocyte trafficking, cytokine release, and antigen presentation in a time-of-day-dependent manner. Misalignment between endogenous rhythms and external cues, known as circadian desynchronization, has been linked to systemic inflammation, loss of immune homeostasis, and increased autoimmune susceptibility. Although several studies suggest that the timing of immunotherapy infusions may affect efficacy and toxicity, the role of circadian factors in immune-mediated neurotoxicity has not been systematically explored [4].

This report describes a fatal case of progressive neuropathy occurring during PD-1 blockade in a patient with advanced melanoma. Retrospective review of laboratory data and infusion timing revealed temporal associations between inflammatory biomarker surges, worsening neurological symptoms, and irregular scheduling of immunotherapy. Based on these observations, the hypothesis of “Chrono-Immune Neuropathy” is proposed, suggesting that disrupted temporal alignment may contribute to the development or amplification of neurologic immune-related adverse events [5, 6].

## Case presentation

A 48-year-old man was diagnosed in June 2022 with a painful pigmented lesion on the right lower leg. Excisional biopsy confirmed BRAF-wild-type malignant melanoma (Breslow III, Clark IV, R0). Initial staging established pT4bN2bM0 stage IIIC disease. In January 2023, he developed right inguinal lymphadenopathy, and lymph node dissection confirmed metastatic melanoma with capsular infiltration.

**Figure 1:**
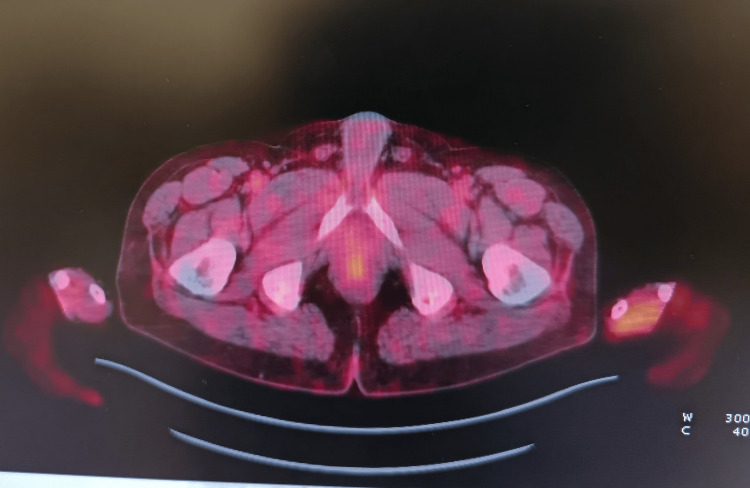
No distant metastases was noted. Mild postoperative FDG uptake in the inguinal region (SUVmax = 2.9), consistent with inflammatory change was seen. FGD: fludeoxyglucose; SUVmax: maximum standard unit value

A baseline PET/CT in July 2022 demonstrated no evidence of distant metastatic disease, with only mild postoperative fludeoxyglucose (FDG) uptake in the inguinal region (maximum standard unit value (SUVmax) = 2.9) (Figure 1). This scan established the radiological baseline prior to initiation of immunotherapy.

**Figure 2:**
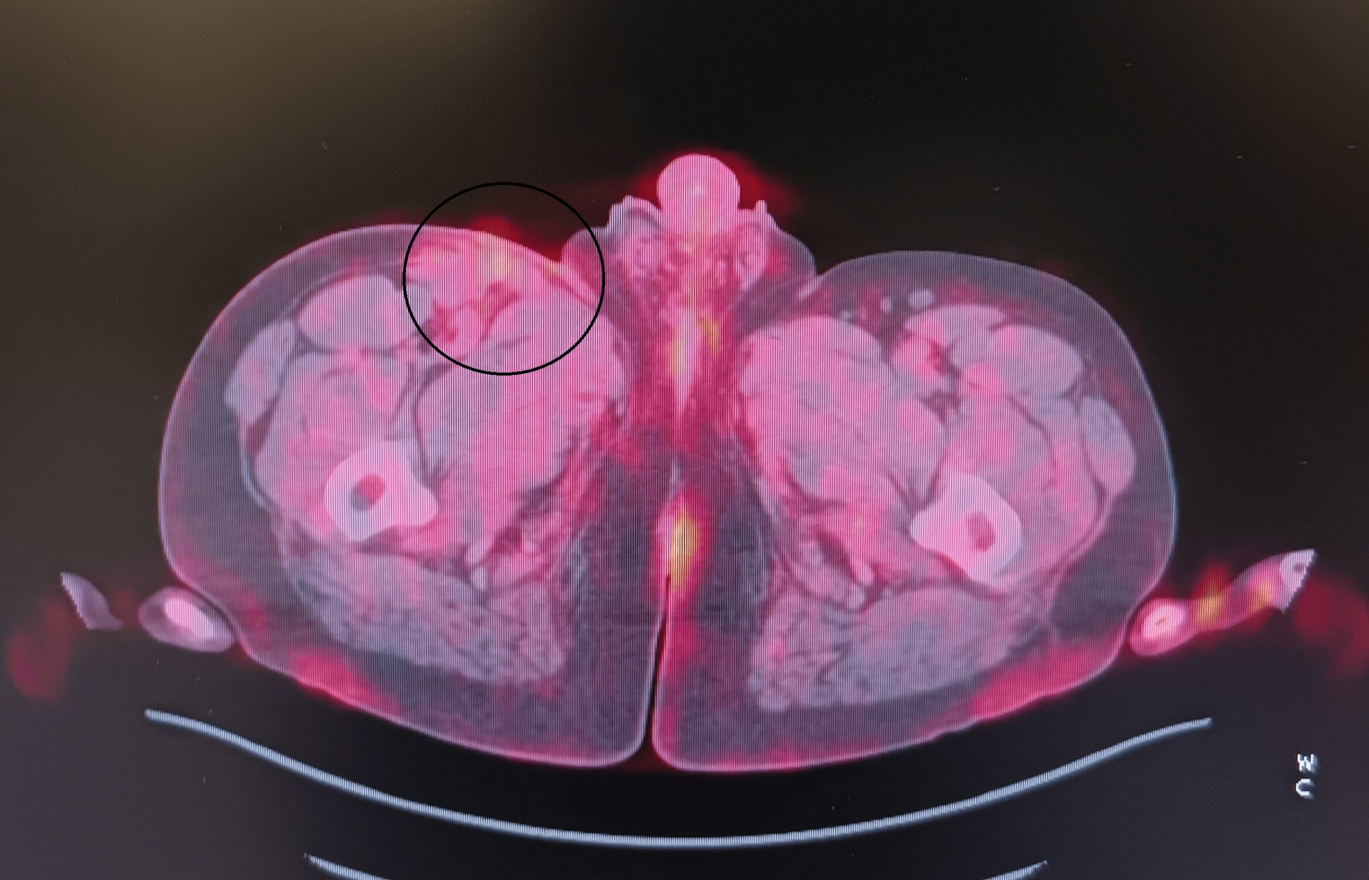
Increasing FDG uptake in the right iliac region, suggested early metabolic progression (SUVmax = 11.64). FGD: fludeoxyglucose; SUVmax: maximum standard unit value

**Figure 3:**
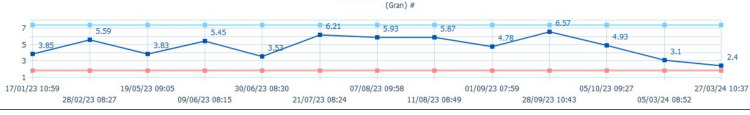
Time-series chart of neutrophil granulocyte counts during pembrolizumab therapy

A follow-up PET/CT in September 2023 revealed metabolically active right iliac lymph nodes (SUVmax 11.64), consistent with progression, and a hypermetabolic lesion in the distal rectum (SUVmax 9.72), interpreted as likely inflammatory. Based on these findings, the diagnosis was revised to pT4bN2bM1 (lym) stage IV disease (Figures 2-3).

The patient started pembrolizumab (200 mg every three weeks) in early 2023. At treatment initiation, he had an Eastern Cooperative Oncology Group (ECOG) performance status of 0 and reported no baseline neuropathic symptoms. Serial hematologic measurements were collected at each treatment visit, and the neutrophil-to-lymphocyte ratio (NLR) was calculated as the absolute neutrophil count divided by the absolute lymphocyte count (granulocytes (Gran)/lymphocytes (Lym)). All values were obtained from routine complete blood count testing during pembrolizumab therapy. Laboratory data from the subsequent nivolumab-ipilimumab phase were not included, as treatment was administered at a different institution and corresponding laboratory datasets were not available.

After several cycles, he developed progressively worsening, refractory neuropathic pain in the lower body, described as burning and stabbing. The pain steadily intensified after each infusion, and escalating analgesic regimens, including oxycodone, buprenorphine, fentanyl (including 50 mcg transdermal preparations), and morphine, failed to provide meaningful relief. His ECOG performance status declined from 1 to 2, accompanied by sleep disturbance and mood worsening.

Due to radiological progression and limited clinical benefit, treatment was escalated to combined ipilimumab-nivolumab in late 2023. Shortly afterward, the patient experienced further deterioration with intensification of neuropathic pain and functional loss. A brain MRI in March 2023 showed no metastases or structural abnormalities. Infectious, metabolic, and paraneoplastic causes were excluded.

For the purposes of longitudinal immune monitoring, the NLR was calculated using all available laboratory data obtained during pembrolizumab monotherapy. Laboratory results from the subsequent nivolumab-ipilimumab phase were not included, as treatment was administered at a different institution and complete datasets were not accessible. Therefore, immune trend analysis reflects the pembrolizumab treatment period only.

**Figure 4:**
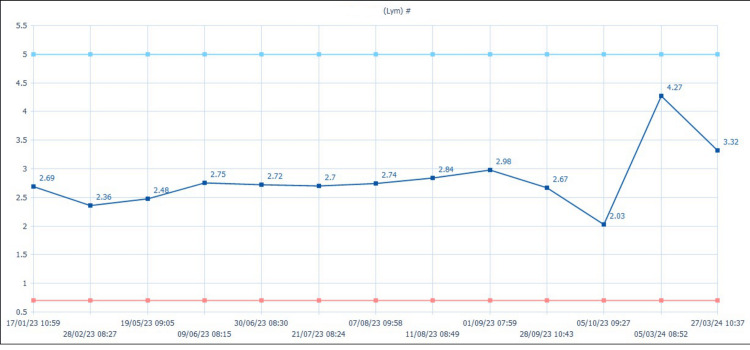
Time-series chart of absolute lymphocyte counts during pembrolizumab therapy; Fluctuations correspond to clinical worsening and contribute to dynamic neutrophil-to-lymphocyte ratio (NLR) variation.

Serial laboratory data demonstrated fluctuations in inflammatory markers, including NLR (ranging from 2.4 to 5.6), platelet-to-lymphocyte ratio (PLR; 110 to 185), and systemic immune-inflammation index (SII). Peaks in these indices coincided with clinical exacerbations of neuropathic pain despite the absence of infection or corticosteroid withdrawal (Figures 3-4).

A retrospective review of infusion records revealed marked inconsistency in the timing of pembrolizumab administration, ranging from 08:15 to 15:00. The highest inflammatory values and worst symptom flares occurred after late-afternoon infusions. Early-morning infusions were associated with relatively milder symptoms (Table 1).

**Table 1:** Temporal dynamics of the neutrophil-to-lymphocyte ratio (NLR) Temporal fluctuations in the NLR demonstrated a distinct oscillatory pattern across treatment. Peaks in NLR preceded neurological deterioration, suggesting a link between inflammatory activation and symptom severity. Gran: granulocytes; Lym: lymphocytes

Date	Gran (×10⁹/L)	Lym (×10⁹/L)	NLR
17-01-2023	3.85	2.69	1.43
28-02-2023	5.59	2.36	2.37
19-05-2023	3.83	2.48	1.54
09-06-2023	5.45	2.75	1.98
30-06-2023	3.52	2.72	1.29
21-07-2023	6.21	2.7	2.3
07-08-2023	5.93	2.74	2.16
11-08-2023	5.87	2.84	2.07
01-09-2023	4.78	2.98	1.6
28-09-2023	6.57	2.67	2.46
05-10-2023	4.93	2.03	2.43
05-03-2024	3.1	4.27	0.73
27-03-2024	2.4	3.32	0.72

Despite escalation of supportive therapy and palliative radiotherapy to the iliac nodes, the patient continued to deteriorate and ultimately died from complications of progressive disease and unresolved neurotoxicity.

## Discussion

Immune-mediated neuropathies are rare but clinically significant immune-related adverse events associated with immune checkpoint inhibitors. Their presentation is heterogeneous and often nonspecific, with variable patterns of pain, sensory disturbances, and weakness that complicate diagnosis and management [7]. Proposed mechanisms include autoreactive T-cell activation, dysregulated cytokine release, neuroglial injury, and disruption of the blood-brain barrier. In this patient, radiological studies revealed no intracranial metastases or structural abnormalities, and infectious, metabolic, and paraneoplastic causes were excluded, supporting an immune-mediated etiology [8,9].

Circadian rhythms are fundamental to immune homeostasis, influencing cytokine secretion, lymphocyte trafficking, and antigen presentation in a time-of-day-dependent manner [10,11]. Growing evidence indicates that the timing of immune checkpoint inhibitor administration can modulate both therapeutic efficacy and toxicity, with morning infusions generally associated with more favorable outcomes. However, while these chronotherapy principles have been described in the context of overall treatment response and common immune-related adverse events, neurologic toxicities remain underrepresented in circadian-oriented analyses [12].

In this case, retrospective analysis revealed a temporal clustering of inflammatory biomarker elevations, worsening neuropathic symptoms, and variability in the timing of anti-PD-1 infusions. Immunologic fluctuations were quantified using serial absolute neutrophil and lymphocyte counts, enabling temporal mapping of NLR dynamics in relation to infusion timing. Laboratory data from the subsequent nivolumab-ipilimumab phase were not included, as treatment was administered at a different institution and complete datasets were unavailable.

Afternoon administrations were temporally associated with higher NLR, PLR, and SII values, alongside clinical exacerbation of neuropathic pain [13-15]. Although causality cannot be inferred from a single case, the parallel presentation of infusion timing, laboratory indices, and symptom worsening suggests that circadian misalignment may contribute to heightened immune activation affecting peripheral nerves [16]. Importantly, unlike dermatologic or endocrine immune-related adverse events, the severity of neurologic toxicities does not consistently correlate with treatment efficacy, underscoring the need for separate mechanistic and temporal consideration [17].

The term “Chrono-Immune Neuropathy” is, therefore, proposed not as a novel concept of chronotherapy, but as a hypothesis-generating clinical framework that applies established chronobiological principles to immune-mediated neuropathy during immune checkpoint blockade [18]. This case illustrates how temporal awareness in treatment delivery may aid in recognizing vulnerability windows for neurologic toxicity. Prospective studies integrating circadian timing into immunotherapy protocols are warranted to further explore whether time-of-day-aligned strategies could improve tolerability and individualization of treatment, particularly in patients at risk of neuroimmune complications [19,20].

## Conclusions

This case illustrates a rare but severe manifestation of immune-mediated neuropathy occurring during PD-1 blockade. In the absence of metastatic, infectious, metabolic, or paraneoplastic causes, the temporal association between inflammatory biomarker surges, worsening neurological symptoms, and variability in the timing of immunotherapy infusions suggests that circadian desynchronization may have contributed to the observed neurotoxicity. Although causality cannot be established from a single case, the convergence of clinical deterioration with disrupted temporal alignment supports the hypothesis of “Chrono-Immune Neuropathy” as a descriptive, hypothesis-generating framework. This observation highlights the potential relevance of consistent, circadian-aware scheduling in immunotherapy and underscores the need for future studies evaluating time-of-day-sensitive treatment strategies to better understand and potentially mitigate immune-related adverse events.
